# Doctor Crane's Letter


**Published:** 1841

**Authors:** 


### DOCTOR CRANE'S LETTER.*

Some person, we know not who, sent us a few weeks since, a New-York newspaper, containing a letter signed J. W. C. This letter, as our readers are, no doubt aware, they having been pretty generally furnished with the paper in which it appeared, asserts that an attack had been made upon its author—*Doctor Crane, Dentist*—by the New-York editor of this journal, in a note appended to an article written by ourself, and that it grew out of a refusal on his part to subscribe to our publication. Doctor Crane unquestionably had a right to refuse to subscribe, and we should not have thought any the less of him for so doing. Had he not been guilty of a most gross and unwarrantable violation of professional courtesy, in charging, not only the gentlemen having the superintendence of this publication in New-York, but also every member of the dental profession, who has not been regularly educated in medicine, with a want of capacity to conduct such a periodical, his name would not have appeared on our pages in the manner in which it did. No importunity of our agent could form a sufficient excuse for such an uncalled-for accusation.

---

* It was not my intention to have noticed the allusions made to me, as the New York Editor of the American Journal of Dental Science, by Dr. Crane, in an article published in some of the New York papers, and industriously circulated among Dentists throughout the country ; and should not do so now, but for the purpose of expressing my high appreciation of the feelings which prompted two gentlemen of the profession to take up the matter, and to place the author of those allusions in his own proper attitude.

When one of the papers containing these allusions, was put into my hands, I read what I supposed to be some editorial remarks ; and not having the pleasure of being acquainted with its editor, I immediately inquired of a friend of his, who is also an editor, what I was to understand by this proceedure ; and I was informed that it was not likely the remarks were in reality editorial, but had been published as such things commonly are, by being paid for as advertisements.

The letter signed by J. W. C., I did not read, nor have I read it to this day ; and indeed, did not know until reading the article above, that it contained the remarks quoted by my associate, and by him so justly commented upon.

I would take this occasion of informing E. N. that the reason why his very handsome letter addressed to the Editor of one of our New-York papers, was not published. The letter was sent to me, with the request to know whether I wished that it should be published, to which I replied, that I did not desire any of my acquaintances or friends, to publish anything in my defence ; but that I was rather pleased with the pains which Dr. C. was taking to make known to the public the estimation in which I hold him. The communication of E. N. I have in my possession ; and shall preserve it as a token of respect and esteem, shewn for me personally by its able author ; and the noble and generous sentiments which it expresses throughout, command my warmest admiration.

N. Y. Editor.

With Doctor Crane personally, we have no acquaintance, nor were we, until quite recently, aware that there was such a person in existence. We therefore, entertain no ill will against him whatever. He may be all he says he is, and we doubt not that he is a man of some cleverness in his profession, and that no one is more sensible of this than himself; but realy we do not recollect to have ever read a *vainer*, *more pompous*, or *egotistical* production in our life; and never were we more fully convinced of the truth of the remarks of St. Real, that, "He who speaks of a *doctor* does not always speak of a *learned man*, but only of a man, who ought to be learned," than while perusing it.

We did not at first think it worthy of notice, but coming as it does from so *learned* a source, a brief review of it may not be uninteresting to our readers—especially as in it, are advanced some new and singular doctrines.

In the first place, the doctor complains, that since the expose of his discourteous conduct about dentists who had not received medical educations, by the New-York editor, gentlemen of the profession had been less inclned to extend to him "the hand of fellowship," and, that many of those who had continued friendly to him, "were not considered worthy of an invitation to the late Dental convention,"—himself, "sharing in the exclusion." The first part of this may, for aught we know, be true, and if so, he ought not to complain. He has nobody to blame but himself, and ought not to expect the hand of fellowship to be extended to him by those of his professional brethren whom he had treated with such gross disrespect. But as regards the reason of his not being invited to attend the "late Dental Convention," in common with gentlemen who may have "remained friendly" to him, we can assure Dr. C., and all who may be under the impression which he seems to have taken up, that the expose of his conduct towards the New-York con ductors of this periodical, was the cause, is wholly incorrect. The members of that convention, knew of many worthy and respectable dentists whom they would have been glad to have had present, but it was thought that few would be likely to attend until something like a nucleus was formed, or in other words, a society regularly organized, and it was on this account that they were not invited.

Having disposed of this part of the letter, we pass over about one half of the paragraph following, to where the author says, "the last conversation I had with him,"—the New-York editor—"was to instruct him on the vitality of the teeth, and that was not the first time he received instruction from the same source." What *egotism* and *vanity!* We wonder if

Doctor Crane supposes that because Dr. P. did him the *favour* to listen to his views on the vitality of the teeth, or any other subject pertaining to dental science, he received any information of which he was not before possessed, and that too very likely before he (Doctor Crane) had thought about entering the profession? But for his vanity, we should be inclined to doubt it; he however, is so liberally endowed with this, that it would not surprise us if he conceited himself to be the only wise man living.

If it be so, will he give us some information on the vitality of teeth, that is not to be found in all the works written and published on that subject? We shall receive it thankfully, and give him full credit for any new information he may communicate.

After this, he boasts of having given lectures to the New-York Dentists' Society, " and giving *them*,"—we suppose he means its members, though he speaks only of the society—" *many new patterns of instruments*," &c. &c. Again, with the greatest self-gratulation, he says—" They," referring to the society, " *have also taken patterns of my operating Chair, which* I INVENTED *about ten years since; and now he*," meaning Dr. P., " *accuses me of not aiding in the improvement and elevation of the science.*" Proceeding with this kind of bombast, he a little further on asks—" *And why have the Medical Students of Crosby-street College invited me to lecture to them on diseases connected with the teeth; and after receiving the lectures gratuitously, sent me business sufficient to pay for a full course!*"

These are truly puzzling questions, and we are certain we do not know why the students of Crosby-street College did all which the Doctor says they did; but if he considered his course of instruction as of much value, and they sent him patients enough to pay for it, we pity them. Good operators can rarely get more than the value of their services, and if Doctor C. can be ranked with this class—and far be it from us to question his claims to skill—he must at any rate be a good *fee-ologist*, to obtain from his patients not only enough to pay him for his services rendered to them, but also, in addition to this, enough to pay him for a full course of lectures. We doubt not that he might do well to deliver a course of lectures on *fee-ology*. That is a department in which a great many are not well informed. We do not ourself profess to be very well versed in it; and should the doctor offer to instruct in this branch, as, by the by, it is a very important one, we might be induced to go on to New-York to avail ourself of the knowledge he must be so eminently qualified to impart on this subject.

Proceeding with his letter, the Doctor in the next place tells us, "*that there are about twenty diseases appertaining to the teeth, which are not investigated by our Medical Faculty in their Lectures.*" Of this we were not before apprized; and although we have read with some attention, the principal works that have been published in England, Ireland, Scotland, France, Germany, and America, on the diseases of the maxillary organs, we do not recollect to have heard that there were that number of diseases that were not treated of by medical Lecturers. A synopsis of them would be highly interesting; and we should be glad if the learned Doctor would favour us with their *nosology*. We feel assured, such a work would be very acceptable to the profession. These diseases not being treated of by medical Lecturers, he says "*The consequence is, the medical man refers his patient to the dentist when such a disease occurs in his practice,*" meaning, we suppose, any of the twenty; "*and, if the dentist has not a medical education, he refers him back to the medical man; and thus the patient falls between them.*" This, we must confess, is a curious kind of logic. If these diseases are not treated on by medical writers or teachers, a medical education could avail the dentist nothing in their treatment; and if he be possessed of knowledge that enables him to do this, he must have obtained it from dental writers, or from the source from whence he received his professional education. It must have constituted a part of this.—If on the other hand, the pathology and therapeutical indications of these diseases, are taught in medical schools, there would be no necessity of the physician's referring patients labouring under any of these maladies, to the dentist. Doctor C. must reason better than this, if he would not have his claims to a superiority of knowledge of the diseases of the buccal and maxillary organs, over many of his brethren, who do not profess to have been regularly educated in medicine, questioned; and others will suppose that there is more of "*shadow,*" than "*suhstance,*" in his pompous pretensions—his M. D. to the contrary, notwithstanding.

We would not only challenge, but defy, this would be profound Doctor, to give some account of any one of the *twenty diseases* spoken of by him, which was not discovered, described, and successfully treated long before he was born, and consequently, before the world became enlightened by his learning, logic, discoveries and inventions, not one word of either of which have we ever heard from any other source than his own letter now under consideration.

We do not argue against the importance of a knowledge of several of the branches of medicine to a skilful practitioner of dental surgery.—This we know to be essential. Our views upon professional qualifications were given at some length in the third number of this periodical, and to which the note that so much excited Dr. C's ire, was appended.

If there are those who wish to know Dr. P's views on this subject, we would refer them to his chapter on the qualifications of the dentist, published in his treatise on the teeth about twenty years ago ; and also to his lecture, delivered before the Society of Dentists, in New-York, on the same subject, published in the 19th number of the United States Medical and Surgical Journal, where the necessity of such knowledge is as strongly urged upon the student as the force of language will permit. For the information of those who have seen the invidious and malicious remarks, so extensively circulated, with the evident design of injuring one who, with those to whom he is professionally known, stands wholly beyond the reach of malice or detraction, I will make a few quotations from the ablest authority in Europe, published twenty years ago, to show that he did obtain that preparatory knowledge, the necessity of which he so earnestly recommends to others ; and but a moiety of which have we yet had any proof that Doctor C—— to this day possesses.

When he can furnish to the profession a better system of practice, and carry it out more successfully than Dr. P—— has done, after a thorough course of professional study and practice, his malicious and envious remarks, addressed to his Boston correspondent, may be entitled to some serious consideration ; but they certainly will not until then.

"An Essay on the Disorders and Treatment of the Teeth. By Eleazar Parmly, Dentist,"

"The author of this little volume, and his brother now in America, we know to be able and ingenious dentists. The present little volume contains perspicuous directions for the Management of the Teeth, and sensible observations on the causes of their decay."

Medico Chirurgical Journal, Edited by James Johnson, M. D., Physician Extraordinary to His late Majesty the King of England.

"We have much pleasure in announcing a very valuable "Essay on the Disorders and Treatment of the Teeth," from the pen of Mr. Eleazar Parmly, a scientific dentist of St. James's Street, London, which is well calculated to put an end to dentifrice quackery. In this little tract Mr. Parmly very ably points out the several duties of a dentist. The author has visited America and France, to collect information on the treatment of the teeth, and has also attended the practice of the most eminent dentists of London, with the same view ; and after giving the different practices a trial, he has detailed those modes of operating in cases of decayed and loose teeth, irregularity in their arrangement, &c., &c., which he is satisfied may be adopted "with prudence and safety."

"We have no hesitation in saying that a perusal of this work will satisfy the reader that it is the production, not only of a skilful dentist, but, what is of no less consequence, an honest man; a man who wishes to communicate the information he has collected at a considerable labour and expense, for the benefit of his fellow creatures."

*Medical Review, page* 1020. *Edited by R. Reece, M. D., London* 1821.

"The instruments hitherto in use for the extraction of teeth, have, to my apprehension, been deficient of that neatness and convenience for adaptation which every operation on the human frame so justly demands. Besides his finished qualifications as a dentist, therefore, it gives me pleasure to notice the merits of Mr. Eleazar Parmly, in contriving an instrument, admirable for its simplicity, which completely sets aside these obvious disadvantages."

"*View of the Structure, functions, aud disorders of the Stomach and Alimentary organs of the human body." By Thomas Hare, F. L. S., &c., Fellow of the Royal College of Surgeons in London*—1821.

After noticing one other passage in the letter under review, we shall dismiss it. The last paragraph commences by saying, "I hear that a medical society has granted him," Dr. P., "the degree of M. D. From what society he received it I have not learned; but one thing we may rely upon—that it was not granted unsought,* nor on account of his knowledge of medicine; nor of his education; nor of his late operations on the teeth." Now, in all these envious inuendoes, Dr. Crane is mistaken. To our knowledge, the honor was conferred without any

* If *Doctor* Crane applies this language to me in his letter, and has given his readers to understand that he *guessed* it was so, I will merely remark that he will be obliged to guess again, and write another letter to his highly favoured Boston correspondent, in order to inform him correctly, because he did not, with all his "cuteness," *guess* right. But if, without a *guess*, he declares it to be true, I should myself be guilty of concealing the truth if I did not pronounce it utterly false—never having in my life, sought, solicited or expected the honor so kindly conferred.

Although in our first Medical Colleges and Schools, a Diploma is gained only by young gentlemen of a high order of talent, yet if I had thought the mere possession of one to hang on the wall of my operating room, as *Doctor* C. has done, necessary to secure professional success, I *believe* that I could have obtained one twenty years ago, where, and in the way, the *Doctor* acquired his—judging from the display of science and learning instanced in the above review, that the terms on which Diplomas are there *sometimes* granted must be exceedingly easy, and within the reach of young men of the most meager, as well as those of the highest capacity. N. Y. Editor.

knowledge or application of Dr. P's. The degree was conferred by a University: the act was voluntary, and on account of *his* "*knowledge in medicine*," his "*education*," and not only *late*, but operations for the last twenty-four or five years, on the teeth; and three other gentlemen of the Dental profession received a like honor, and for like reasons, from the same University. Being in possession of these facts, we think it no more than right that they should be made public, inasmuch as the remarks of Dr. Crane are calculated to produce a false impression among those with whom the New-York editor of this Journal may not be acquainted. BALT. ED.]

